# Impact of mild cortisol excess on osteoporosis and the mediating role of sarcopenia-related traits: A Mendelian randomization study

**DOI:** 10.1097/MD.0000000000044153

**Published:** 2025-08-29

**Authors:** Junyu Zhu, Shijie Zhou, Mingqi Jiang, Wenying Wang, Jun Yan

**Affiliations:** a Department of Orthopedics, Third Medical Center of Chinese People’s Liberation Army General Hospital, Beijing, China; b Department of Orthopedics, The Daping Hospital, Chongqing, China; c Department of Orthopedics, The First People’s Hospital of Yunnan Province, Kunming, Yunnan, China.

**Keywords:** bone mineral density, cortisol, femoral neck, Mendelian randomization, osteoporosis, sarcopenia-related traits

## Abstract

Studies show excessive cortisol is linked to osteoporosis (OP). However, the impact of mild cortisol excess (MCE) on bone mineral density (BMD) remains unclear. Sarcopenia may play a key role in this, particularly in aging or stress contexts. To investigate the association between MCE and OP outcomes and the proportion of this association that is mediated through SP using Mendelian randomization (MR). MR study using summary statistics with traits were obtained from publicly available genome-wide association studies (GWAS) of OP and sarcopenia-related traits. Three single-nucleotide polymorphisms (SNPs) associated with plasma cortisol concentrations in the CORtisol NETwork consortium were used as instrumental variables. All participants were of European ancestry. Random-effect inverse-variance weighted (IVW) method was used as the main analysis of MR, and a series of sensitivity analyses were performed to detect heterogeneity and horizontal pleiotropy. A 2-step MR approach was used to investigate whether the mediating pathway from MCE to OP was mediated by sarcopenia-related traits. Cortisol increases OP risk in the femoral neck (FN, OR = 0.874, 95% CI: 0.786–0.973, *P* = .0138), while reducing it in the lumbar spine (LS, OR = 1.140, 95% CI: 1.008–1.291, *P* = .037) and heel (OR = 1.027, 95% CI: 1.000–1.054, *P* = .047). The mediation analysis via 2-step MR showed that sarcopenia mediates up to 8.4% of the cortisol-induced osteoporosis risk in the LS. This MR analysis suggests that MCE primarily increases OP risk in the FN, rather than affecting the body systemically. It also shows that SP mediates up to 8.4% of the cortisol-induced OP risk in the lumbar spine, highlighting the importance of muscle-strengthening exercises in preventing OP.

## 1. Introduction

Osteoporosis (OP) is a multifactorial metabolic bone disorder characterized by a decrease in bone mineral density (BMD) and an increased risk of fractures.^[1–4]^ Projections indicate that by 2025, OP will be responsible for approximately 3 million fractures annually in the United States (US). In the European Union (EU), it is estimated that the annual osteoporotic fracture rate will increase by 25% between 2019 and 2034, and with their aging population this is set to increase.^[5,6]^

It has been reported that the onset and progression of OP are closely related to the regulation of endocrine hormone secretion.^[7–10]^ Cortisol, as a stress hormone, plays a crucial role in the onset and progression of OP and is closely linked to our daily lives.^[11,12]^ Thus, elucidating the relationship between cortisol and OP has positive clinical significance for the prevention of OP.

It is well established that pathological levels of cortisol have been extensively studied and reported for their detrimental effects on bone density and bone tissue.^[13]^ However, the relationship between mild cortisol excess (MCE) and OP remains controversial.

BMD measured by dual X-ray DXA is the current reference standard for the diagnosis of OP.^[14]^ Some studies indicate that MCE may not necessarily result in reduced BMD, and that the decline in BMD is not directly proportional to the severity of the disease.^[15]^ This stands in contrast to the conclusions drawn by other studies.

It may be attributed to other mediating factors. Research indicates that MCE may accelerate the progression of SP, a condition closely associated with the development of OP.^[16–19]^ It suggests that SP may be one of the key mediating pathways through which cortisol impacts the development of OP. However, uncertainty remains concerning the causal influence of MCE on OP outcomes and the role of SP as a mediating pathway. Better characterization of the association between MCE and OP risk would have significant public health implications for reducing the burden of this disease.

Mendelian randomization (MR) is a valid approach for causal inference using genetic variants as instrument variables (IVs), which can effectively overcome the confounding bias of traditional epidemiological studies.^[20]^ Our MR study had 2 primary aims: first, to assess the causal relationship between MCE and OP; And second, to evaluate the role of SP as a mediating pathway in the association between MCE and OP. Specifically, we primarily focused on BMD at 4 common osteoporotic fracture sites: lumbar spine BMD (LS-BMD), femoral neck BMD (FN-BMD), forearm BMD (FA-BMD), heel BMD (H-BMD) and total BMD (T-BMD).^[21,22]^ For the mediating factors, we included sarcopenia-related traits.^[23,24]^

## 2. Methods

### 2.1. Study design

**Figure 1.**
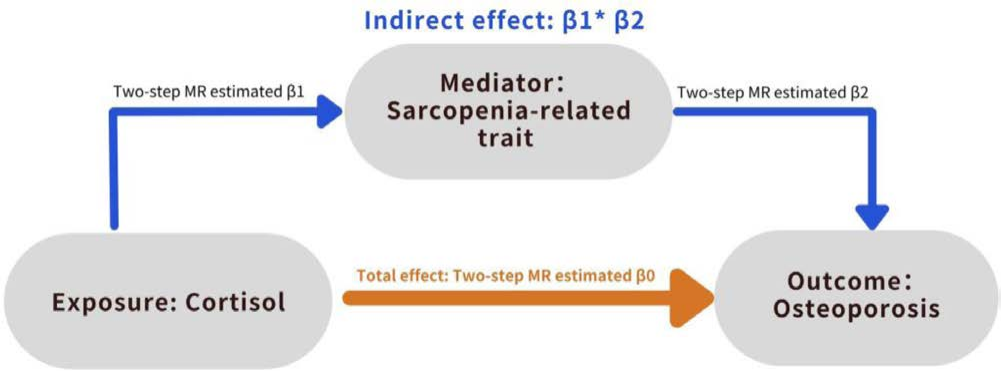
Schematic presentation of the Mendelian randomization design.

We used a 2-sample MR design: a genetic IV analysis based on summary-level data with SNPs as instruments for the risk factor. For causal estimates from MR studies to be valid, 3 assumptions must be adhered to: the genetic variants are highly associated with the exposure, the genetic variants are not associated with any potential confounder of the exposure–outcome association, and the variants exclusively affect the outcome through the exposure.^[25]^ We first performed a 2-sample bidirectional MR to investigate the causal relationship of MCE (measured as cortisol) with OP (measured as BMD) risk. Subsequently, we utilized MR analysis to investigate the causal relationship between MCE and sarcopenia-related traits (measured as body lean mass and low grip strength). A 2-step MR analysis was conducted to examine whether SP mediated the relationship between MCE and OP, given the significant causal associations between MCE and SP, and their respective causal effects on OP (Fig. 1).

This study was reported in accordance with the guidelines of strengthening the reporting of observational studies in epidemiology using MR: the STROBE-MR statement.^[26]^ And the summary-level data from genome-wide association studies (GWAS) were used to find genetic proxies of an exposure and investigate the associations of these proxies with an outcome. All studies had been approved by a relevant ethical review board and participants had given informed consent, which did not require institutional review board approval because of the public characteristics of the data of GWAS.

### 2.2. Instrumental variable selection for cortisol

Cortisol variants were drawn from a GWAS meta-analysis of 12,597 individuals in the CORtisol NETwork (CORNET) consortium, comprising 11 Western European population-based cohorts.^[27]^ The study excluded participants using glucocorticoids, pregnant or breastfeeding women, and twins. Plasma cortisol concentrations were measured via immunoassay from blood samples collected between 7:00 and 11:00 AM (mean: 472 nmol/L). The data were standardized (mean = 0, SD = 1) and log-transformed for analysis.^[27]^ Plasma cortisol concentrations were measured between 7:00 and 11:00 AM to align with the natural circadian rhythm of cortisol, which typically peaks in the early morning and gradually declines throughout the day. This standardized timing helps minimize inter-individual variability and reduces potential confounding from external factors that may influence cortisol levels later in the day. Based on recent MR studies of cortisol, we selected 3 SNPs independently associated with plasma cortisol concentrations at genome-wide significance (*P* < 5e−08, *r*^2^ < 0.3). The selection of 3 SNPs was guided by recent findings from GWAS. While incorporating a larger number of SNPs could potentially enhance precision, the 3 selected variants represent the most robust and independent instruments currently available. This approach is supported by prior MR studies and reflects a balance between maximizing the genetic explanatory power for cortisol levels and minimizing the risk of horizontal pleiotropy.^[27,28]^ The variants rs12589136 and rs11621961 are located in the SERPINA6 gene, which encodes corticosteroid-binding globulin (CBG). The SNP rs2749527, located in the SERPINA1 gene encoding α1-antitrypsin, was adjusted for age, sex, and ancestry principal components, explaining 0.54% of the variation in plasma cortisol levels^[28]^ (Table 1). The *F*-statistics for IVs suggested that genetic variants strongly predicted plasma cortisol concentrations (*F*-statistics > 10).^[29]^

**Table 1:** Overview of genetic data sets.

Trait	GWAS ID	Year	PMID	No. sample size
Cortisol	ieu-a-1012	2014	25010111	12,597
LS-BMD	ieu-a-982	2015	26367794	28,498
FN-BMD	ieu-a-980	2015	26367794	32,735
FA-BMD	ieu-a-977	2015	26367794	8143
Heel-BMD	ebi-a-GCST006979	2019	30598549	426,824
T-BMD	ebi-a-GCST005348	2018	29304378	56,284
WBLM	ukb-b-13354	2018	NA	454,850
ALM	ebi-a-GCST90000025	2020	33097823	450,243
Right-hand grip	ukb-b-10215	2018	NA	461,089
Right-hand grip	ukb-b-7478	2018	NA	461,026

ALM = appendicular lean mass, FA-BMD = forearm bone mineral density, FN-BMD = femoral neck bone mineral density, GWAS ID = genome-wide association studies identity number, Heel-BMD = heel bone mineral density, LS-BMD = lumbar spine bone mineral density, NA = not applicable, PMID = PubMed identification number, T-BMD = total-bone mineral density, WBLM = whole body lean mass.

### 2.3. Outcomes

The GEnetic factors for osteoporosis consortium (GEFOS, http://www.gefos.org) is a large international cooperation organization, involving many famous research groups. LS-BMD, FN-BMD, and FA-BMD data were obtained from the 2015 GWAS summary statistics on BMD in a population of European ancestry.^[21]^ The GWAS summary statistics for Heel-BMD from the UK Biobank (UKB).^[30]^ 426,824 individuals of European ancestry identified SNPs associated with Heel-BMD. The GWAS summary statistics for T-BMD were derived from a 2018 meta-analysis, which included a population with 86% of European ancestry^[22]^ (Table 1).

### 2.4. Mediators

For sarcopenia-related traits, we selected whole body lean mass (WBLM), appendicular lean mass (ALM), and grip strength. Grip strength, as a measure of muscle strength, and WBLM, as a measure of muscle mass, are both well-established predictors of sarcopenia. ALM, primarily determined by skeletal muscle, serves as an additional measure of muscle mass and enhances the predictive accuracy for SP when combined with other metrics. The GWAS summary statistics for hand grip strength were obtained from the UKB, which includes 461,089 United Kingdom (UK) individuals for right-hand grip strength, and 461,026 individuals for left-hand grip strength.^[24]^ The GWAS summary statistics for WBLM (*N* = 454,850) from the UKB.^[24]^ The ALM-related values were quantified by the sum of fat-free mass with 450,243 UKB cohort participants (https://www.ebi.ac.uk/gwas/publications/33097823)[23] (Table 1).

### 2.5. Statistical analysis

All analyses were run using R, version 4.3.2 (R program for statistical computing) and the R packages MR, version 0.10.0, and 2 sample MR, version 0.6.4. The inverse-variance weighted (IVW) method was used as the main approach in this MR analysis, which provides accurate estimates in the absence of heterogeneity and directional pleiotropy between the cortisol and SP.^[31–33]^ The heterogeneity of the IVW model was assessed by Cochran *Q* test. If Cochran *Q* test suggested significant heterogeneity (*P* < .05), we turned from the fixed IVW model to the random-effects model. MR-Egger analysis was conducted to estimate the causal effect, with the intercept used to interpret and assess both directional and horizontal pleiotropy. A significant deviation of the intercept from zero (*P* < .05) suggests the presence of directional pleiotropy.^[25,34]^ The odds ratio (OR) was used as the measure of association in the MR analysis. The OR reflects the change in the odds of osteoporosis per unit increase in plasma cortisol levels or sarcopenia-related traits. A significant OR would suggest a causal relationship, while a non-significant OR would imply no discernible causal effect of cortisol or sarcopenia on osteoporosis risk. The MR pleiotropy residual sum and outlier (MR-PRESSO) method was used to evaluate horizontal pleiotropy.^[35]^ If horizontal pleiotropy is detected, it is corrected by removing outliers and examining whether significant differences in the causal estimates exist before and after outlier removal. In order to evaluate the stability of the results, an array of sensitivity analyses was executed. A Leave-one-out analysis was initiated to scrutinize whether a single SNP was the driving force behind the causal signal.^[36]^

### 2.6. Mediation analysis

If the methods above confirm a causal relationship between cortisol and OP without evidence of directional pleiotropy, a 2-step MR approach will be employed. This method will assess the mediating role of SP in the causal pathway between the exposure (cortisol) and the outcome (OP), while estimating the magnitude of the mediation effect.^[37]^

## 3. Results

Based on the GWAS of European ancestry, we identified a total of 3 independent SNPs that were associated with the plasma cortisol concentrations and OP, as well as 3 independent SNPs associated with the cortisol and SP.

### 3.1. The causal effect of cortisol on OP

**Figure 2.**
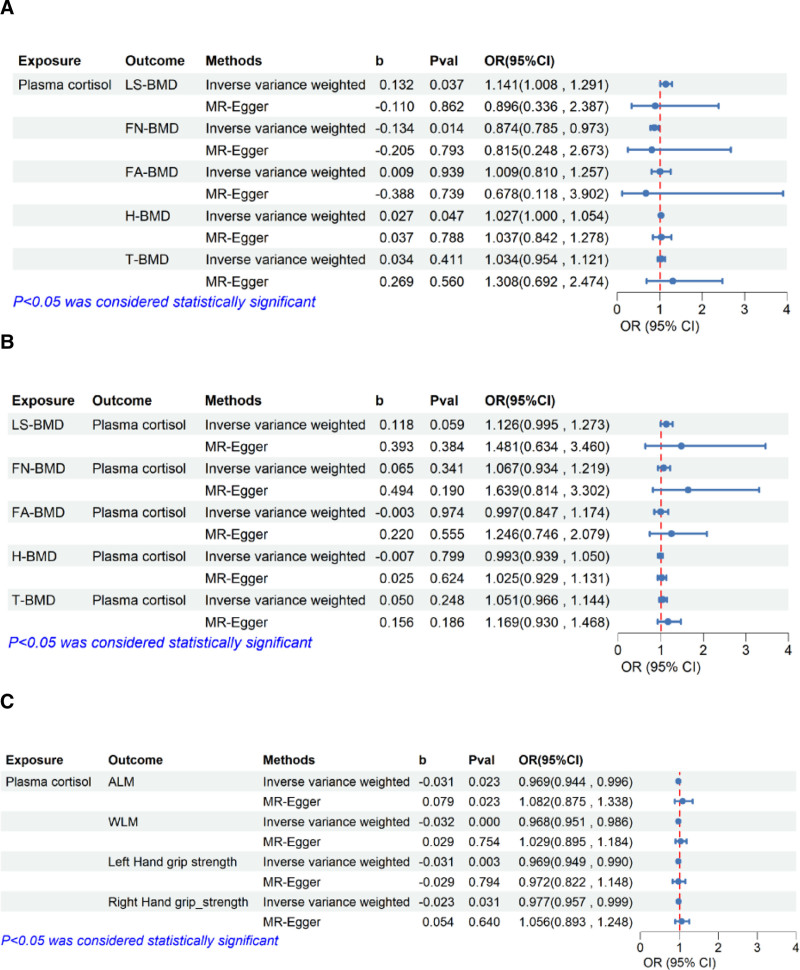
Assessing the causal relationship between cortisol levels, osteoporosis, and sarcopenia. (A) and (B) The causal relationship between cortisol levels and osteoporosis. (C) The causal relationship between cortisol levels and sarcopenia.

In IVW MR analysis, each 1 SD increase in plasma cortisol concentrations was associated with increased risk OP in the FN region (OR = 0.874, 95% CI: 0.786–0.973, *P* = .014), contrasted with a decreased risk in the LS (OR = 1.140, 95% CI: 1.008–1.291, *P* = .037) and heel regions (OR = 1.027, 95% CI: 1.000–1.054, *P* = .047). However, elevated plasma cortisol levels showed no significant causal association with OP in the FA (*P* = .939) or the whole body (*P* = .411) (Fig. 2A). Furthermore, no reverse causal relationship was found between elevated plasma cortisol levels and the occurrence or progression of OP. There was no evidence of heterogeneity (Cochran *Q*, *P* > .05) and no indication of pleiotropy in the MR-Egger analysis (MR-Egger intercept *P* > .05) (Fig. 2B; Fig. S1, Supplemental Digital Content, https://links.lww.com/MD/P784, Tables S1 and S2, Supplemental Digital Content, https://links.lww.com/MD/P785).

### 3.2. The causal effect of cortisol on SP

We subsequently examined the impact of cortisol on sarcopenia-related traits. The MR analysis revealed that the elevated plasma cortisol levels were significantly associated with reductions in WBLM (OR = 0.968, 95% CI: 0.951–0.986, *P* < .001), ALM (OR = 0.969, 95% CI: 0.944–0.996, *P* = .023), left grip hand strength (OR = 0.969, 95% CI: 0.949–0.990, *P* = .003) and right hand grip strength (OR = 0.977, 95% CI: 0.957–0.997, *P* = .030). There was no evidence of heterogeneity (Cochran *Q P* > .05) and no indication of pleiotropy in the MR-Egger analysis (MR-Egger intercept *P* > .05) (Fig. 2C; Fig. S2, Supplemental Digital Content, https://links.lww.com/MD/P784; Tables S1 and S3, Supplemental Digital Content, https://links.lww.com/MD/P785).

### 3.3. The causal effect of SP on OP

**Figure 3.**
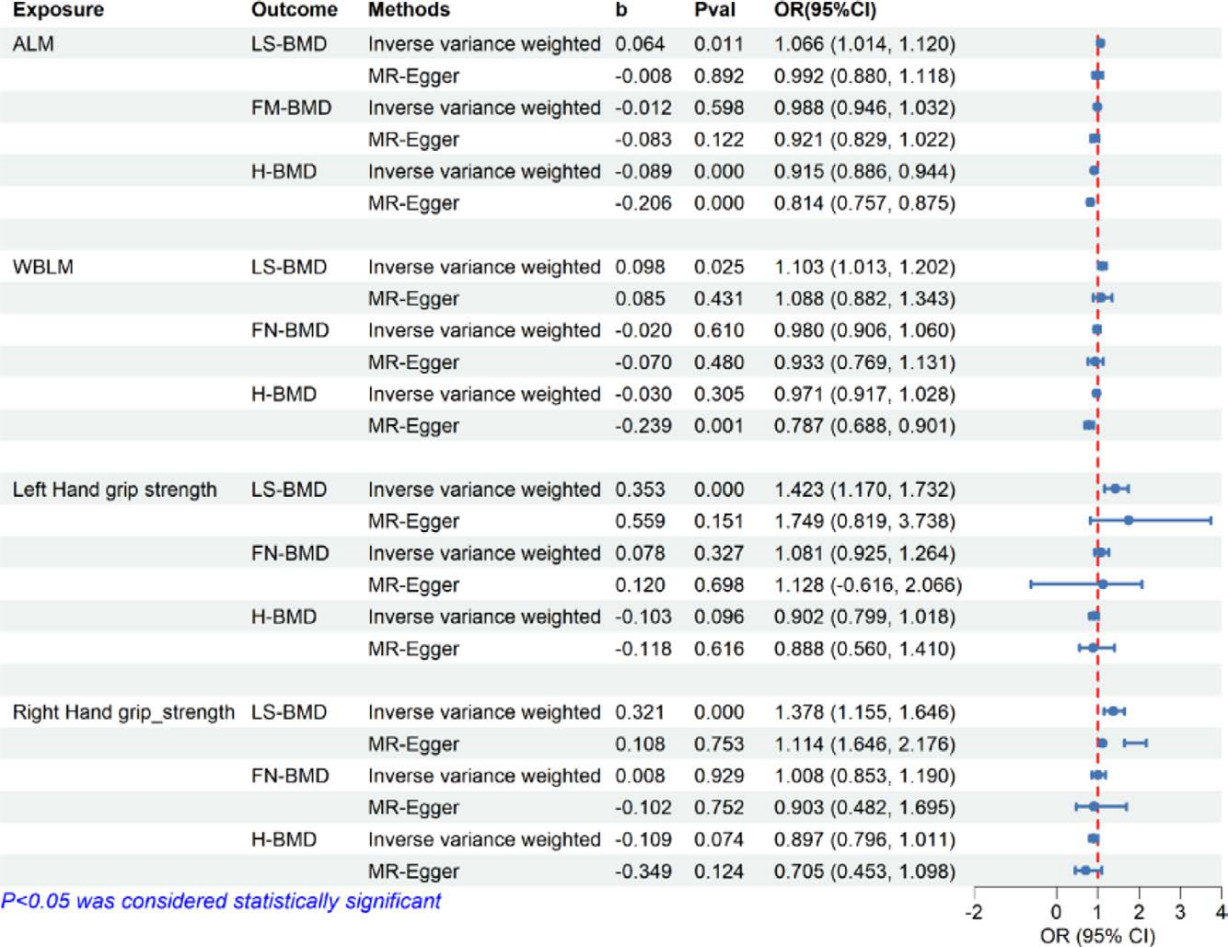
Assessing the causal relationship between sarcopenia-related traits and osteoporosis.

We extracted SNPs that were strongly associated with sarcopenia-related traits, defined as *P* < 5 × 10^−8^, and that were independent of one another, defined as a pairwise *R*^2^ < 0.01 based on the GWAS summary statistics. Next, we excluded the 3 independent SNPs previously identified as being associated with plasma cortisol concentrations and SP. Finally, according to result obtained from the causal relationship between cortisol and SP, we identified independent SNPs associated with SP and OP (LS-BMD, FN-BMD, H-BMD). Through MR analysis, we identified that the reduction in WBLM (OR = 1.103, CI: 1.013–1.202, *P* = .025), ALM (OR = 1.066, CI: 1.015–1.120, *P* = .011), left grip hand strength (OR = 1.423, CI: 1.170–1.732, *P* < .001) and right hand grip strength (OR = 1.378, CI: 1.155–1.646, *P* < .001) is significantly associated with a increased risk osteoporosis in LS. However, there is no causal relationship between sarcopenia-related traits and an increased risk of OP in the FN and heel (ALM-FN(P) = 0.598, WBLM-FN(P) = 0.610, left grip hand strength-FN(P) = 0.327, right hand grip strength-FN(P) = 0.929, ALM-H(P) = 0.000, WBLM-H(P) = 0.305, left grip hand strength-H(P) = 0.096, right hand grip strength-H(P) = 0.074). MR-Egger analysis detected no evidence of pleiotropy in the aforementioned results (ALM-LS(P) = 0.892, WBLM-LS(P) = 0.431, left grip hand strength-LS(P) = 0.151, right hand grip strength-LS(P) = 0.753, ALM-FN(P) = 0.122, WBLM-FN(P) = 0.480, left grip hand strength-FN(P) = 0.698, right hand grip strength-FN(P) = 0.752). But the heterogeneity test showed significant heterogeneity among selected IVs (*P* < .05) (Fig. 3, Tables S1, S4–S6, Supplemental Digital Content, https://links.lww.com/MD/P785).

### 3.4. Mediation analysis

The mediation effects of sarcopenia-related traits on the relationship between cortisol and lumbar OP are as follows: ALM was 0.064 (95% CI: −0.005–0.001, proportion: −1.5%), WBLM was 0.098 (95% CI: −0.012–0.005, proportion: −2.4%), left grip hand strength was 0.353 (95% CI: −0.080–0.058, proportion: −8.4%), right grip hand strength was 0.321 (95% CI: −0.064–0.049, proportion: −5.6%) (Table 2).

**Table 2:** Associations between genetically estimated cortisol and osteoporosis outcomes after accounting for sarcopenia-related traits as mediators.

Mediator	Total effect	Direct effect	Mediation effect (95% CI)	Mediated proportion (%)
ALM	0.132	0.134	0.064 (−0.005, 0.001)	−1.5
WBLM	0.132	0.135	0.098 (−0.012, 0.005)	−2.4
Left-hand grip strength	0.132	0.143	0.353 (−0.080, 0.058)	−8.4
Right-hand grip strength	0.132	0.139	0.321 (−0.064, 0.049)	−5.6

The results of the mediation analysis and the proportion mediated are shown.

ALM = appendicular lean mass, WBLM = whole body lean mass.

## 4. Discussion

This MR study indicates a significant causal relationship between cortisol levels and the progression of OP in specific regions, including the FN and heel. Notably, elevated cortisol levels do not exhibit a potential causal associated with a reduction in T-BMD. It suggests that MCE may be primarily associated with the development and progression of localized OP. Moreover, it also indicate the substantial mediating role of sarcopenia between cortisol and lumbar OP.

The focus on OP is one of the initiatives that science and societies look for to address the social and health problems exacerbated by the global population aging. It is well known that OP, the most prevalent bone metabolic disorder regulated by multiple hormones, significantly impacts global public health by increasing fracture risks, particularly among post-menopausal women and the elderly.^[38]^ Hormones play crucial roles in the development and progression of OP.^[39]^ Understanding the relationship between each hormone and OP is essential for the prevention and treatment of OP. However, no randomized controlled trials have yet been conducted to investigate the relationship between a specific hormone and OP while fully controlling for the potential confounding effects of other hormones. We selected SNPs with specific effects on the SERPINA6/SERPINA1 locus, which influences CBG, as IVs. CBG, the primary binding protein for glucocorticoids, transports cortisol to target tissues, thereby regulating the availability of biologically active free cortisol in those tissues. Furthermore, these SNPs influence the genetic variability of plasma total cortisol concentrations, which to some extent reflects the potential activity of cortisol itself.^[40]^ Leveraging genetic variants as robust IVs and controlling for the influence of other hormones, our study offers novel insights, suggesting that MCE is a significant potential factor in promoting OP progression in the FN. In contrast, MCE may help to prevent the onset and progression of OP in the LS and heel. This observation seems to be at variance with previous evidence and established conclusions. This may be attributed to MCE primarily impairing trabecular bone architecture and reducing overall bone quality, rather than directly affecting BMD.^[7]^

More importantly, our study demonstrated the mediating role of sarcopenia-related traits and quantified the proportions mediated by SP between MCE and lumbar OP. The research conducted by Mazziotti et al demonstrated that the risk of fractures is significantly elevated in patients with CD, with vertebral bodies being the most frequently affected skeletal sites.^[8]^ Based on this study, we can reasonably speculate that this may be due to the LS’s increased susceptibility to the effects of SP. A study indicated that SP is a potential risk factor for acute osteoporotic vertebral fractures. This also indirectly supports the conclusions drawn from this study.^[41]^ This reflects the importance of enhancing muscle training in the prevention of osteoporosis. It provides guidance for preventing osteoporosis in our daily lives through a healthy lifestyle.

A major strength of our study is that we applied instrumental variable analyses using genetic instruments that allowed for assessment of the causal role of SP mediators in the association between Cortisol and OP. Due to the random allocation of alleles and because these alleles are static throughout an individual’s life, this design is much less likely to be affected by nondifferential measurement error of the mediator and confounding compared with traditional observational studies.^[42,43]^ This was further supported by our sensitivity analyses that did not indicate any bias due to pleiotropy.

This study also has some limitations. First, this study was based on morning plasma cortisol concentrations by immunoassay. Secondly, some of the individuals included in the GWAS of cortisol, grip strength, and lean mass were nonelderly; thus, this may be a potential sampling bias. The study focused primarily on individuals of European ancestry, which may limit generalization to other populations, highlighting the need for genomic studies in diverse ancestral groups. The MR framework relies on a key assumption that the risk conferred by an exposure is equivalent whether mediated by genetics or environment, and that genetic risk is conferred through the exposure of interest rather than via pleiotropic effects. Moreover, in the 2-sample MR analysis of SP and T-BMD, the inclusion of populations with different ancestries may introduce bias into our final results. Finally, we selected 3 SNPs as IVs for cortisol based on *r*^2^ < 0.3, among which there may be a small degree of linkage disequilibrium. When SNPs in linkage disequilibrium are used as independent IVs, the genetic effects on cortisol may be overestimated. Thus, rigorous clinical and fundamental research is needed in the future to provide more robust evidence for our conclusions.

## 5. Conclusion

This MR study suggests that MCE may be involved in the development of region-specific OP, particularly affecting the FN. Notably, MCE showed no significant association with generalized osteoporosis. SP mediates the development of osteoporosis in the spine. Therefore, our research further elucidates the underlying mechanisms by which MCE affects OP and highlights the importance of strengthening muscle training in the prevention of osteoporosis.

## Author contributions

**Methodology:** Shijie Zhou.

**Software:** Mingqi Jiang.

**Writing – original draft:** Junyu Zhu, Jun Yan.

**Writing – review & editing:** Wenying Wang, Jun Yan.

## Supplementary Material


