# Supplementary material for: Impact of mild cortisol excess on osteoporosis and the mediating role of sarcopenia-related traits: A Mendelian randomization study
Source: Medicine (Baltimore). 2025 Aug 29;104(35):e44153. doi: 10.1097/MD.0000000000044153 (PMC12401307; doi:10.1097/MD.0000000000044153)
Supplement: Supplementary file 1 [file medi-104-e44153-s001.docx]

**eFigure 1.**

**
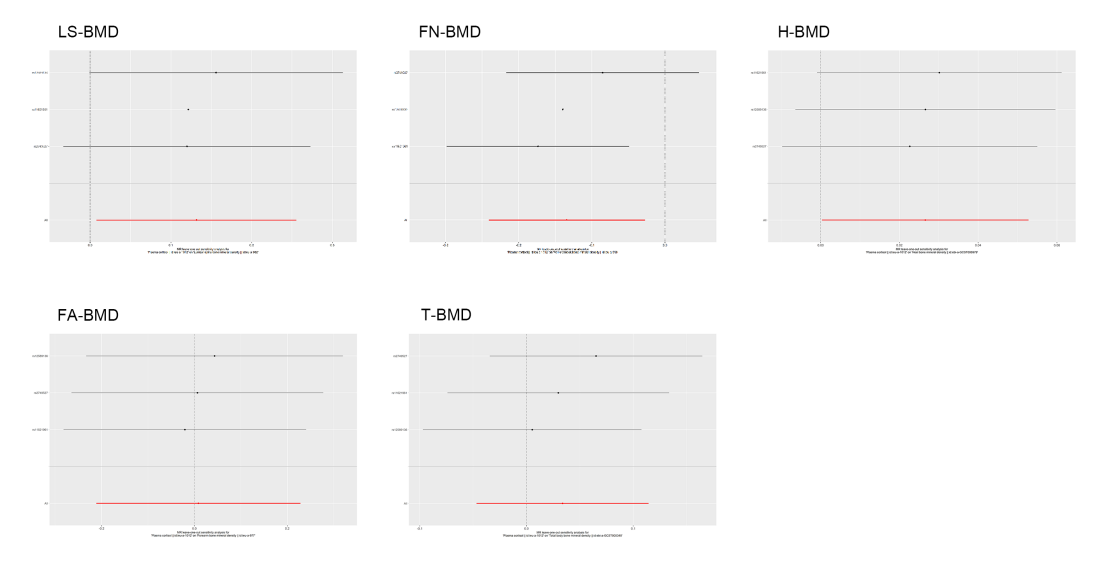
**

In leave-one-out analysis, each individual SNP is sequentially excluded from the inverse

variance weighted MR model to identify outlier SNPs that may drive outsize effects. FA BMD, Forearm bone mineral density; FN BMD, Femoral neck bone mineral density; LS BMD, Lumbar spine bone mineral density; T BMD, Total body bone density.

**eFigure 2.**

**
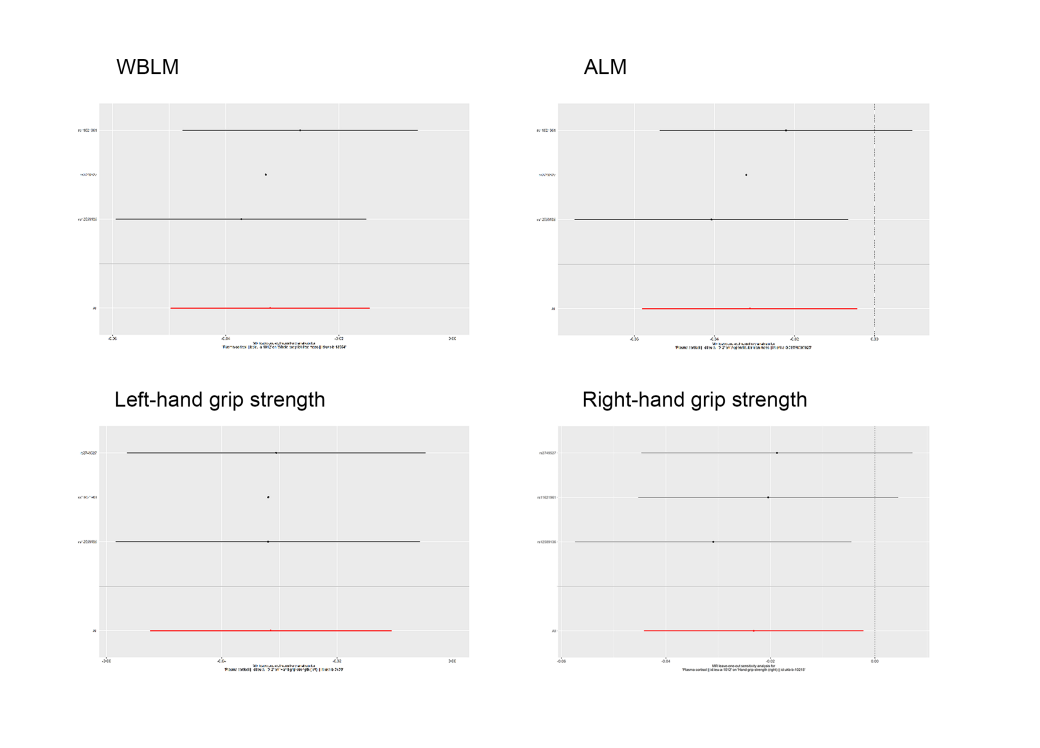
**

In leave-one-out analysis, each individual SNP is sequentially excluded from the inverse

variance weighted MR model to identify outlier SNPs that may drive outsize effects. WBLM, whole body lean mass; ALM, appendicular lean mass.
