# Supplementary material for: Impact of mild cortisol excess on osteoporosis and the mediating role of sarcopenia-related traits: A Mendelian randomization study
Source: Medicine (Baltimore). 2025 Aug 29;104(35):e44153. doi: 10.1097/MD.0000000000044153 (PMC12401307; doi:10.1097/MD.0000000000044153)
Supplement: Supplementary file 2 [file medi-104-e44153-s002.docx]

**eTable 1.**

| **Exposure** | **Outcome** | **Method** | **OR(95%CI)** | **P-value** |
| --- | --- | --- | --- | --- |
| Cortisol | LS-BMD | Inverse variance weighted | 1.141(1.008,1.291) | 0.036 |
| Cortisol | WBLM | Inverse variance weighted | 0.968(0.951,0.986) | 3×10^-4^ |
| Cortisol | ALM | Inverse variance weighted | 0.969(0.944,0.996) | 0.023 |
| Cortisol | Left-hand grip strength | Inverse variance weighted | 0.968(0.949,0.990) | 0.003 |
| Cortisol | Right-hand grip strength | Inverse variance weighted | 0.977(0.957,0.998) | 0.031 |
| WBLM | LS-BMD | Inverse variance weighted | 1.103(1.01E+00,1.20E+00) | 2.46E-02 |
| ALM | LS-BMD | Inverse variance weighted | 1.066(1.015,1.120) | 1.11E-02 |
| Left-hand grip strength | LS-BMD | Inverse variance weighted | 1.423(1.170,1.73E+00) | 4.20E-04 |
| Right-hand grip strength | LS-BMD | Inverse variance weighted | 1.378(1.155,1.64E+00) | 3.82E-04 |

LS BMD, Lumbar spine bone mineral density; WBLM, whole body lean mass; ALM, appendicular lean mass

**eTable 2.**

| **Exposure** | **Outcome** | **Cochran Q statistic** | **Heterogeneity P-value** | **MR-Egger Intercept** | **Intercept p-value** |
| --- | --- | --- | --- | --- | --- |
| Plasma cortisol | LS-BMD | 0.243 | 0.885 | 0.021 | 0.711 |
| Plasma cortisol | FA-BMD | 0.221 | 0.895 | 0.034 | 0.732 |
| Plasma cortisol | T-BMD | 1.227 | 0.541 | -0.020 | 0.599 |
| Plasma cortisol | FN-BMD | 17.496 | 0.231 | -0.028 | 0.243 |
| Plasma cortisol | H-BMD | 0.235 | 0.889 | -0.001 | 0.939 |

FA BMD, Forearm bone mineral density; FN BMD, Femoral neck bone mineral density; LS

BMD, Lumbar spine bone mineral density; T BMD, Total body bone density.

**eTable 3.**

| **Exposure** | **Outcome** | **Cochran Q statistic** | **Heterogeneity P-value** | **MR-Egger Intercept** | **Intercept p-value** |
| --- | --- | --- | --- | --- | --- |
| Plasma cortisol | WBLM | 0.973 | 0.615 | -0.005 | 0.546 |
| Plasma cortisol | ALM | 1.324 | 0.516 | 0.010 | 0.492 |
| Plasma cortisol | Left-hand grip strength | 0.014 | 0.993 | 2×10^-4^ | 0.978 |
| Plasma cortisol | Right-hand grip strength | 0.896 | 0.639 | -0.007 | 0.529 |

WBLM, whole body lean mass; ALM, appendicular lean mass

**eTable 4.**

| **Exposure** | **Outcome** | **Cochran Q statistic** | **Heterogeneity P-value** | **MR-Egger Intercept** | **Intercept p-value** |
| --- | --- | --- | --- | --- | --- |
| WBLM | LS-BMD | 759.415 | 1.16E-13 | 1×10^-4^ | 0.890 |
| WBLM | FN-BMD | 868.129 | 4.31E-23 | 7×10^-4^ | 0.585 |
| WBLM | H-BMD | 8572.639 | 0.000 | 0.003 | 8×10^-4^ |
| ALM | LS-BMD | 880.402 | 8.71E-16 | 0.002 | 0.194 |
| ALM | FN-BMD | 914.553 | 1.52E-18 | 0.002 | 0.145 |
| ALM | H-BMD | 8825.123 | 0.000 | 0.003 | 5×10^-4^ |
| Left-hand grip strength | LS-BMD | 270.175 | 1.87E-09 | -0.002 | 0.582 |
| Left-hand grip strength | FN-BMD | 232.153 | 7.40E-06 | -5×10^-4^ | 0.888 |
| Left-hand grip strength | H-BMD | 2511.967 | 0.000 | 2×10^-4^ | 0.946 |
| Right-hand grip strength | LS-BMD | 265.092 | 2.62E-07 | 0.003 | 0.519 |
| Right-hand grip strength | FN-BMD | 317.224 | 1.35E-12 | 0.001 | 0.725 |
| Right-hand grip strength | H-BMD | 2841.165 | 0.000 | 0.003 | 0.271 |

FA BMD, Forearm bone mineral density; FN BMD, Femoral neck bone mineral density; LS BMD, Lumbar spine bone mineral density; TB BMD, Total body bone density; WBLM, whole body lean mass; ALM, appendicular lean mass

**eTable 5.**

| **Exposure** | **Outcome** | **MR-PRESSO Global test P-value** | **Method** | **OR(95%CI)** | **P-value** |
| --- | --- | --- | --- | --- | --- |
| WBLM | LS-BMD | <0.050 | Inverse variance weighted | 1.105(1.014,1.204) | 0.022 |
| WBLM | FN-BMD | <0.050 | Inverse variance weighted | 0.983(0.908,1.064) | 0.672 |
| WBLM | H-BMD | <0.050 | Inverse variance weighted | 0.988(0.927,1.053) | 0.706 |
| ALM | LS-BMD | <0.050 | Inverse variance weighted | 1.067(1.015,1.121) | 0.022 |
| ALM | FN-BMD | <0.050 | Inverse variance weighted | 0.034(0.948,1.035) | 0.665 |
| ALM | H-BMD | <0.050 | Inverse variance weighted | 0.930(0.898,0.963) | 6.23E-05 |
| Left-hand grip strength | LS-BMD | <0.050 | Inverse variance weighted | 1.412(1.159,1.721) | 6×10^-4^ |
| Left-hand grip strength | FN-BMD | <0.050 | Inverse variance weighted | 1.075(0.920,1.258) | 0.362 |
| Left-hand grip strength | H-BMD | <0.050 | Inverse variance weighted | 0.926(0.831,1.031) | 0.162 |
| Right-hand grip strength | LS-BMD | <0.050 | Inverse variance weighted | 1.378(1.152,1.648) | 5×10^-4^ |
| Right-hand grip strength | FN-BMD | <0.050 | Inverse variance weighted | 0.995(0.842,1.175) | 0.949 |
| Right-hand grip strength | H-BMD | <0.050 | Inverse variance weighted | 0.913(0.784,1.063) | 0.242 |

FA BMD, Forearm bone mineral density; FN BMD, Femoral neck bone mineral density; LS BMD, Lumbar spine bone mineral density; TB BMD, Total body bone density; WBLM, whole body lean mass; ALM, appendicular lean mass

**eTable 6.**

| **Exposure** | **Outcome** | **Cochran Q statistic** | **Heterogeneity P-value** | **MR-Egger Intercept** | **Intercept p-value** |
| --- | --- | --- | --- | --- | --- |
| WBLM | LS-BMD | 752.258 | 2.15E-13 | 3×10^-4^ | 0.827 |
| WBLM | FN-BMD | 862.195 | 2.11E-23 | 7×10^-4^ | 0.542 |
| WBLM | H-BMD | 6629.861 | 0.000 | 0.003 | 0.006 |
| ALM | LS-BMD | 878.930 | 5.82E-16 | 0.002 | 0.190 |
| ALM | FN-BMD | 910.205 | 1.34E-18 | 0.002 | 0.158 |
| ALM | H-BMD | 6979.289 | 0.000 | 0.003 | 5×10^-4^ |
| Left-hand grip strength | LS-BMD | 266.377 | 2.44E-09 | -0.002 | 0.691 |
| Left-hand grip strength | FN-BMD | 228.994 | 8.24E-06 | -3×10^-4^ | 0.954 |
| Left-hand grip strength | H-BMD | 904.590 | 2.70E-129 | -0.002 | 0.414 |
| Right-hand grip strength | LS-BMD | 265.088 | 1.53E-07 | 0.003 | 0.522 |
| Right-hand grip strength | FN-BMD | 307.783 | 5.19E-12 | 0.001 | 0.756 |
| Right-hand grip strength | H-BMD | 2084.513 | 0.000 | 0.004 | 0.291 |

FA BMD, Forearm bone mineral density; FN BMD, Femoral neck bone mineral density; LS BMD, Lumbar spine bone mineral density; TB BMD, Total body bone density; WBLM, whole body lean mass; ALM, appendicular lean mass
